# Giving Birth in Unpredictable Conditions: Association between Parents’ COVID-19 Related Concerns, Family Functioning, Dyadic Coping, Perceived Social Support and Depressive Symptoms

**DOI:** 10.3390/healthcare10122550

**Published:** 2022-12-16

**Authors:** Theano Kokkinaki, Katerina Koutra, Olga Michopoulou, Nicole Anagnostatou, Lina Chaziraki, Panagiota Kokarida, Eleftheria Hatzidaki

**Affiliations:** 1 Child Development and Education Unit, Laboratory of Applied Psychology, Department of Psychology, University of Crete, 741 50 Rethymnon, Greece; 2 Addiction Psychology Laboratory, Department of Psychology, University of Crete, 741 50 Rethymnon, Greece; 3 Department of Neonatology/Neonatal Intensive Care Unit, University Hospital of Heraklion, School of Medicine, University of Crete, 715 00 Heraklion, Greece; 4 Department of Obstetrics—Gynecology, General Hospital of Chania, 733 00 Chania, Greece; 5 Department of Psychology, University of Crete, 741 50 Rethymnon, Greece

**Keywords:** COVID-19 pandemic, postpartum period, family functioning, dyadic coping, perceived social support, depressive symptoms

## Abstract

Background: The way postpartum parents’ COVID-19-related concerns are associated with the family environment, support resources and depressive symptoms areunder-investigated. Methods: Two hundred and forty-three new parents (132 mothers, 111 fathers) completed self-report questionnaires within an 8-week period after birth. Parental concerns for COVID-19-related life changes were assessed with the COVID-19 Questionnaire, perceived social support with the Multidimensional Scale of Perceived Social Support, perceived family functioning with the Family Adaptability and Cohesion Evaluation Scales IV Package, dyadic coping behaviors with the Dyadic Coping Inventory and maternal/paternal postnatal depression with the Edinburgh Postnatal Depression Scale. Results: (a) Higher levels of COVID-19-related concerns about daily life were associated with lower levels of family communication, satisfaction and increased depressive symptomatology in both parents, and with lower levels of family functioning in mothers; (b) Maternal health care COVID-19-related concerns were linked with lower levels of family communication, lower perceived social support and with an increase in maternal depressive symptoms; and (c) COVID-19-related concerns about neonate hospitalization were associated with increased maternal depressive symptoms. Conclusion: Τhese findings suggest that COVID-19-related concerns had a common negative effect on both postpartum mothers’ and fathers’ mental health and on certain aspects of family functioning.

## 1. Introduction

The COVID-19 pandemic constitutes a global health crisis and, at the same time, a worldwide traumatic experience [1,2]. In Greece, the pandemic outbreak took place while the country was emerging from a long-lasting period of economic and social crisis [3]. Though Greece had a relatively successful response to the first pandemic wave (26 February 2020 to 31 May 2020, restrictions were imposed in the first national lockdown (23.03.20–4.05.20) and continued during the second, third and fourth wave [4,5]. Similarly, to the rest of the world, the pandemic in Greece posed increased COVID-19-related fear, and, combined with the lockdown policies, led to occupational/financial instability and educational disruption, as well as threatened interpersonal relationships and adversely affected the mental health of Greeks [6,7].

A growing public health concern is the potential impact of the SARS-CoV-2 pandemic on high-risk populations, such as pregnant and postpartum women [8,9,10,11]. The transition to parenthood constitutes a major life event for the expectant parent which influences personal, relational, and family well-being [12] and increases the risk of experiencing psychological and relational problems [13]. In the course of the COVID-19 pandemic, pregnant women experienced a prenatal trauma which exacerbated uncertainty and anxiety with health-related concerns and changes to traditional perinatal care. Perinatal experience in the pandemic was complicated by limitations in access to mental health services andunderestimation of the psychological impact of the pandemic on pregnancy. As a consequence, stress and vulnerability of postpartum parents may be amplified during an already demanding life period [14,15].

Restricted evidence from studies in different regions on the way the COVID-19 pandemic affected parentsin the perinatal period (pregnancy to 12 months postpartum) indicatedthat: (a) In the US, women’s worries about their infant contracting SARS-CoV-2were connected with concern aboutthemselvesor their partners contracting the virus [16]; (b) In Portugal, most postpartum mothers reported normal or mild levels of depressive symptomatology [9]. On the contrary, pregnant women and new mothers in Italy experienced high levels of depressive symptoms [17]. Similarly, in the UK, expectant mothers also showed an increased prevalence of depression [15]. In Israel, either mothers’ and fathers’ parental distress did not differ [18], or fathers reported a higher increase in stress during the pandemic [19]. In contrast, in Portugal, mothers reported higher levels of depression compared to fathers [13]; (c) Regarding family relationships, in Portugal and in the US, both increased verbal arguments as well asmore quality time with the partner have been reported [12,13]. In China, paternal perinatal depression risk was negatively associated with family function [20]; (d) In Israel, higher level of marital satisfaction wererelated to lower parenting stress [19]. Portuguese mothers reported higher levels of perceived social support than fathers [13], although they only communicated with a small network of friends/family. In the UK, higher perceived social support was associated with lower anxiety [15], and in the US, decreased perceived social support was associated with poor mental health [21].

Sociodemographic factors, such as parental age, educational level and occupational status, have been associated with parental mental health [22,23,24]. Furthermore, parental age has been related to family relationships [25] and low socio-economic status has been associated with more stressful family life situations [26]. In addition, parental mental health is related to gestational age [27], with maternal depression increasing the risk of prematurity. Furthermore, parental mental health is related to type of delivery [28]; women who delivered byemergency cesarean section reported symptoms of depression at 6 months postpartum more frequentlythan women who had given birth naturally.

Limited research in Greece indicates that the overall levels of parents’ (In the samples of reported studies, parents represent 48.53–71% while the child’s age has not been reported.) depression were lower than the rates in the literature. Similarly to other regions, the mixed picture of the pandemic impact on family relationships and the proportionately larger negative pandemic effect on mothers compared to fathers is confirmed [7,29,30].

The impact of the pandemic on individual mental health and relational functioning may be explained through the Circumplex Model of Marital and Family Systems [31,32,33] and the Systemic-Transactional Model [34,35,36]. According to the first model, perceived family functioning has been described through three dimensions. Family cohesion is related to the emotional bond that members in the family have towards one another. Levels of cohesion range from disengaged to separated (that is, from very low–low to moderate), to connected and to enmeshed (moderate to high–very high). Family adaptability is associated with the amount of change in its leadership, role relationships and relationship rules. Levels of flexibility range from rigid to structured (very low–low to moderate) to flexible and to chaotic (moderate to high–very high). Balanced levels of cohesion (separated, connected) and balanced levels of flexibility (structured, flexible) contribute to effective family functioning and optimal individual’s development. Family communication is related to family members’ skills regarding listening and speaking, sharing feelings, clarity, continuity tracking, respect and regard. Lastly, family satisfaction reflects the satisfaction of all above three dimensions (cohesion, flexibility and communication) [33].

According to Bodenmann’s Systemic-Transactional Model [34,35], the birth of a child may cause dyadic stress. In cases of prolonged stress, people seek out support resources, such as dyadic coping and social support. Dyadic coping refers to the partners’ common appraisals of stress, communication of concerns and engagement in shared coping efforts through positive supportive communication processes. Dyadic coping reduces partners’ distress and is associated with relationship satisfaction [34,37].

Τhis study sought to contribute to a growing body of knowledge by investigating the relationship between COVID-19-related concerns, thefamily environment, dyadic coping, perceived social support and depressive symptoms of postpartum parents in Greece. The family environment may affect the interaction of negative emotions among its members under a crisis condition [31]. Support resources are important for mitigating the effects of stress on individual and relational well-being [38]. Furthermore, perinatal parental psychopathology is related to behavioral, biological, and neurophysiological correlates of infant psychological functioning and may constitute a risk to child development beyond the early years [39].

The objectives of this study were the following:

(a) Describe and compare postpartum mothers’ and fathers’ COVID-19-related concerns, perceived family environment, dyadic coping, perceived social support and depressive symptoms.
(b) Examine the association between dimensions of family functioning, dyadic coping, perceived social support and depressive symptoms for mothers and fathers in the pandemic period.
(c) Identify the association between maternal/paternal pandemic-related concerns and aspects of family functioning, dyadic coping, perceived social support and postpartum depression,
(d) Investigate the independent contribution of each dimension of parental COVID-19-related concerns on the four outcomes (family functioning, dyadic coping, perceived social support and depressive symptoms) after taking into account a variety of potential confounding variables.

## 2. Study Design andProcedure

Participants were recruited through the Department of Neonatology/Neonatal Intensive Care Unit of the University Hospital of Heraklion, and the Department of Obstetric-Gynecology of the General Hospital of Chania in Crete, Greece. Data collection was carried out during the third and fourth pandemic waves (February–September 2021). The infection rate of the fourth wave was 144 times larger than the first wave [5].Inclusion criteria were as follows: (a) both parents had to be Greek citizens; and (b) both parents had to be older than 20 years.

Participants completed scales in the following order: sociodemographic and socioeconomic data, the COVID-19 Questionnaire, the Multidimensional Scale of Perceived Social Support, the Family Adaptability and Cohesion Evaluation Scale, the Dyadic Coping Inventory and the Edinburgh Postnatal Depression Scale.Permission for the use of each psychometric scale was granted by the research team who translated and adapted the instruments in the Greek population. Before every series of questions, a short introduction informing the participants about the questionnaire’s scope was provided. Participants were asked to complete a number where applicable, or tick the box or circle corresponding to their chosen answer. Questionnaires were completedbefore new mothers’hospital discharge, or within an 8-week period after giving birth.

This study received approvals both from the Institutional Review Board and the Scientific Councils of the above hospitals (see Institutional Review Board Statement). All participants were informed about the aim and the procedure of the study and gave their written consent. Participation was anonymous, voluntarily and not compensated.

## 3. Measures

### 3.1. COVID-19-Related Concerns’ Questionnaire

Parental concerns for COVID-19-related life changes were measured according to a 14-item questionnaire which was developed for this study. Participants were asked specifically about COVID-19-related concerns regarding: (a) daily life, such as personal/family health, economic and employment status, lack of social services, changes in eating habits, concentration problems and deterioration of previous health problems (7 items); (b) maternal health care during pregnancy, such as the impact of the pandemic on maternal care services and on the possibility of feeling fear as a result of restrictions in paternal presence during visitation to the obstetrician (2 items); and (c) neonate hospitalization (if applicable), such as the implications the pandemic had on neonate care in the course of delivery/birth, on parental visitation to NICU, the impact of visitation restrictions on maternal mental health before and after birth, and on breastfeeding (5 items). Responses for all items ranged from ‘never’ (=1) to ‘very often’ (=5). In this study, Cronbach’s alpha coefficient for the three factors ranged from 0.59 to 0.73. 

### 3.2. Perceived Social Support

The Greek version of the Multidimensional Scale of Perceived Social Support [40,41,42] (MSPSS) was used for the measurement of perceived social support from close relations such asfriends, family and significant others. MSPP is a 12-item reliable and valid self-rating scale. Ιtems are rated on a seven-point Likert scale ranging from ‘very strongly disagree’ (=1) to ‘very strongly agree’ (=7). The total score ranges from 12 to 84. Higher scores indicate higher levels of perceived social support. In the current study, the scale was found to have good reliability levels (α = 0.93).

### 3.3. Perceived Family Functioning

The Family Adaptability and Cohesion Evaluation Scales IV Package [32,33] (FACES IV) was used to assess perceived family functioning. The FACES IV Package contains the six scales from FACES IV (42 items) [two balanced scales (balanced cohesion, balanced flexibility) and the four unbalanced scales (Disengaged and Enmeshed for cohesion and Rigid and Chaotic for flexibility)], the Family Communication Scale (FCS) and the Family Satisfaction Scale (FSS) (62 items in total). Each family member rates his/her agreement or disagreement with how well each item describes his/her family. Higher scores on the balanced scales are indicative of healthier functioning, and the converse holds true for the unbalanced scales. Three additional ratio scores can be calculated with FACES IV (Cohesion, Flexibility, and Total Circumplex). When each score of the Cohesion and Flexibility ratios is at one and higher, the family system has more balanced levels of cohesion and flexibility. When the Total Circumplex ratio is one or higher, the family system is viewed as more balanced and functional. FACES IV has been translated and validated for the Greek population [43], and has demonstrated good psychometric properties. In the current study, Cronbach’s alpha coefficients for the six subscales ranged from 0.52 to 0.74.

The FCS [33], a 10-item scale, was used to assess many of the most important aspects of communication in a family system. A higher score on the scale indicates more positive communication in a family system. In this study, the Cronbach’s alpha coefficient was 0.87. The FSS [33] is, also, a 10-item scale that appraises the satisfaction of family members in regard to family cohesion, flexibility and communication. A higher score on the scale indicates greater satisfaction in the family system. In this study, the Cronbach’s alpha coefficient was 0.90.

### 3.4. Dyadic Coping

The Greek version of the Dyadic Coping Inventory [44,45,46] (DCI) was used to measure dyadic coping behaviors. The DCI is a 37-item reliable and valid instrument designed to measure dyadic coping. Items are rated on a 5-point scale from ‘very rarely’ (=1) to ‘very often’ (=5). The total DCI score is a sum of items 1 through 35 after reverse coding of negatively keyed items (7, 10, 11, 15, 22, 25, 26 and 27). Items 36 and 37 are not included in the total score. In this study, the Cronbach’s alpha coefficient was 0.90.

### 3.5. Maternal and Paternal Postnatal Depression

The Greek version of the Edinburgh Postnatal Depression Scale [47,48] (EPDS) was used for the measurement of maternal and paternal postnatal depression. EPDS [41] is a 10-question self-rating scale which has been validated for use in mothers and fathers with good psychometric properties. The EPDS identifies mothers/fathers at risk of perinatal depression. Participants were asked to rate the frequency with which they experienced symptoms of depression in the last 7 days. Higher scores reflect a higher presence of depressive symptoms. In this study, Cronbach’s alpha coefficient was 0.83 (0.85 for males and 0.80 for females).

## 4. Statistical Analysis

Descriptive statistics were obtained for the demographic variables. Means and standard deviations, as well as frequencies and proportions, were calculated for continuous and categorical variables, respectively. Cronbach’s alpha coefficient was used to measure the internal consistency of the scales. A student’s t-test or ANOVA was used to evaluate bivariate associations between normally distributed continuous dependent variables and categorical independent variables. Pearson’s r correlation coefficient was used to estimate the strength of the association between continuous dependent and independent variables. The independent contribution of each dimension of parental concern for COVID-19 life changes on the four outcome scales (FACES-IV package, DCI, MSPSS, EPDS) were examined through multivariate linear regression models. Potential confounders (e.g., parental age, educational level, occupational status, type of delivery, and gestational age) were included a priori in the multivariable models. Estimated associations are described in terms of β-coefficients and their 95% confidence intervals (CI). All hypothesis testing was conducted assuming a 0.05 significance level and a two-sided alternative hypothesis. The statistical data analysis was performed by statistical package IBM SPSS Statistics (version 26).

## 5. Results

### 5.1. Participants–Sociodemographic and Socioeconomic Characteristics

Table 1 presents parental and child characteristics of the study population. For this study, 284 families were approached and 243 parents participated (85.5% participation rate). Our sample consisted of 132 mothers (54.3%) and 111 fathers (45.7%). The mean age of mothers and fathers was 32.94 (SD = 5.76) and 36.60 (SD 6.67) years, respectively. A total of 59.1% of mothers had a high educational level (university or Master’s degree), while 45.5% of fathers had completed secondary education. A total of 73.5% percent of mothers were living in urban areas and 97.0% were married.

### 5.2. Descriptive Statistics for COVID-19-Related Life Changes, Family Functioning, Dyadic Coping Social Support and Depressive Symptoms

Table 2 presents the mean scores for the psychosocial variables for the whole sample split by gender. The mean scores for the six subscales of FACES-IV ranged from 15.03 (SD = 4.20) (Disengaged) to 30.05 (SD = 2.79) (Balanced Cohesion). The FACES-IV score for total circumplex ratio has a mean of 1.75 (SD = 0.58), suggesting that participants generally perceived their families as functioning relatively well. The mean scores for the Communication and Satisfaction subscales of FACES-IV were 42.23 (SD = 5.18) and 39.73 (5.86), respectively. The mean scores for dyadic coping and social support were 143.44 (15.25) and 74.00 (SD = 8.70), respectively. Regarding depressive symptoms, the mean scores were 7.90 (SD = 5.23). A minority of mothers (4.5%) and fathers (0.9%) scored ≥13 in EPDS. These scores suggest that participants generally perceived dyadic coping and social support relatively well and depressive symptoms were low-midrange for the majority of postpartum parents. A statistically significant difference between fathers and mothers was found on postnatal depressive symptoms. Specifically, mothers reported higher postnatal depressive symptoms as compared to fathers. As far as COVID-19-related life changes are concerned, the mean scores for the three subscales of the COVID-19 questionnaire were in the midrange, while there were non-significant differences between fathers and mothers. 

### 5.3. Correlations between the Outcome Variables

All FACES dimensions were positively correlated between each other in both mothers and fathers. Furthermore, all FACES-IV dimensions were positively associated with dyadic coping and social support in both parents. In addition, cohesion was negatively correlated with depressive symptoms only in mothers. Communication was negatively correlated with depressive symptoms only in fathers. The other three dimensions—flexibility, total family functioning and communication—were negatively associated with depressive symptoms in both parents. Dyadic coping was significantly associated with social support and depressive symptoms in both parents. Social support and depressive symptoms were significantly correlated in fathers, but not in mothers.

### 5.4. Association between Parental COVID-19-Related Concerns, Family Functioning, Dyadic Coping/Perceived Social Support and Postnatal Depression in Postpartum Mothers and Fathers

According to Table 3, parental concern for COVID-19-related life changes in terms of daily life was negatively associated with both parents’ communication and satisfaction scores and positively with their depressive symptoms. Furthermore, daily life concerns were correlated negatively with maternal reports on cohesion, flexibility and total family functioning, and positively with maternal depressive symptoms. Paternal concerns for COVID-19-relatedchanges in maternal health care were negatively associated with fathers’ reported flexibility and family functioning. In addition, maternal health care concern was associated negatively with maternal perceived social support and positively with maternal depressive symptoms. Finally, parental concern for COVID-19-related life changes in terms of neonate hospitalization was positively associated with maternal depressive symptoms. 

### 5.5. Multivariate Associations of COVID-19-Related Life Changes with Family Functioning, Dyadic Coping, Perceived Social Support and Depressive Symptoms in Parents

The results from the multivariate analysis are presented in Table 4. Participant age, educational level, occupational status, type of delivery and gestational age were included in the multivariable models as potential confounders in the relationship between COVID-19-related life changes with family functioning, dyadic coping, perceived social support and depressive symptoms in parents. The results indicated that higher levels of COVID-19-related concerns about daily life were associated with lower levels of family communication and satisfaction in parents, and lower levels of cohesion, flexibility and total family functioning in mothers. Furthermore, higher levels of concern for COVID-19-related life changes in daily life were related with increased depressive symptomatology in both parents (β coefficient 0.43, 95% CI: 0.24, 0.62 and β coefficient 0.27, 95% CI: 0.10, 0.45 for fathers and mothers, respectively). COVID-19-related concerns about maternal health care were linked with lower levels of family communication, lower perceived social support, and with a 0.48 unit increase in maternal depressive symptoms (β coefficient 0.48, 95% CI: 0.10, 0.86). Finally, COVID-19-related concerns about neonate hospitalization were associated with a 0.45 unit increase in maternal depressive symptoms (β coefficient 0.45, 95% CI: 0.16, 0.74).

## 6. Discussion

The current study aimed to provide a comprehensive examination of the relationship between COVID-19-related concerns, the family environment, social support, dyadic coping and postnatal depression of postpartum mothers and fathers in Crete, Greece. 

We showed that maternal and paternal COVID-19-related concerns were in the midrange. Our evidence is not in accordance withfindings coming from Italy, which show that parents were very worried about the COVID-19-related situation [1].This variation may be attributed to the differentiated impact of the pandemic on the two countries [49] and to the different data collection period between the two studies (March–April 2020, February–September 2021). We indicated that mothers and fathers in the postpartum period did not differ in COVID-19-related life changes concerns. Given that 97% of the sample was married, the similarity between maternal and paternal COVID-19-related concerns may be explained by the fact that, after the birth of a child, both new mothers and fathers experience similar changes in their daily lives and couples’ common experiences mutually influence one another due to their shared interdependence [50,51,52]. Parents had relatively high levels of perceived family functioning, dyadic coping and perceived social support, while depressive symptoms were low to midrange for the majority of them. These may be due to the fact that in Crete, family ties play a crucial role in the lives of people [53].

We showed that mothers reported higher postnatal depressive symptoms compared to fathers. This confirms relevant findings showing higher levels of maternal versus paternal postpartum depression during the pandemic [13]. This finding may be attributed to the fact that the perinatal period is a time of high vulnerability for maternal mental health. During the pandemic, etiological mechanisms for the increasing prevalence of maternal postpartum depression include the fear of being infected and vulnerability of the newborn to the unknown virus and the possible ways of transmission. Public health policies, COVID-19 associated socio-economic consequences (unemployment, additional child care, household responsibilities) and inequalities may have been an additional burden for postpartum mothers [54,55].

COVID-19-related concerns about personal/family health and employment/economic status were negatively correlated only with maternal, reports on cohesion, flexibility and total family functioning, and were positively related with maternal depressive symptoms. This finding confirms the evidence showing that the higher perceived threat from COVID-19 has been associated with increased odds of depression of women in the perinatal period [56,57]. Gender variations in the way concerns about employment status were related with perceived family functioningmay be attributed to differences between men and women in the effects of workplace characteristics on family cohesion. Work and job characteristics influence women’s perceptions of family cohesion [58]. Lockdowns and teleworking caused family–work conflict and led to lower work satisfaction and efficiency for women than men [59]. Furthermore, under the ongoing COVID-19 pandemic, women seem to be more worried about their loved ones and the family, which may interfere in their perceived family functioning, while men are more occupied with effects on economic issues [60] In connection to this, the correlation between paternal COVID-19-related concerns about employmentand postpartum depression may be explained by the association between unstable employment status and postnatal depression in fathers [61]. 

Moreover, higher levels of COVID-19-related life changes were associated with lower levels of family communication and satisfaction in both parents. This finding is consistent with evidence showing that postpartum mothers’ and fathers’ health concerns and COVID-19-related restrictive measures contributed to relationship difficulties [13]. This may be due to the spillover effects of external stress into one’s relationship, decreasing the effective communication with negative effects on relational well-being [35]. The common correlation for mothers and fathers between COVID-19 concerns and both communication and satisfaction are further supported by the identification of satisfaction-to-communication and communication-to-satisfaction associations between spouses [62]. Furthermore, regarding fathers, the negative association between COVID-19 concerns and communication may be related to the negative correlation between communication with depressive symptoms. As for the mothers, the negative association between COVID-19 concerns and satisfaction may be related to the negative correlation between depressive symptoms and satisfaction. This is reinforced by evidence showing that maternal postpartum depression predicts low couple relationship [63]. These findings invite further investigation. 

COVID-19-related concerns of postpartum mothers about their health care were linked with lower levels of family communication, lower perceived social support and with an increase in maternal depressive symptoms. This is in accordance with evidence showing that women in the perinatal period reported decreased social support during the COVID-19 pandemic which was associated with poorer mental health [21]. Therelationship between maternal health care concerns with family communication, perceived social support and depressive symptoms may be connected to pandemic-related health care policies resulting in prohibitions in companionship for antenatal and postnatal tests, and for birth and postnatal visiting. It may be that the pandemic transformed birth experience from being a ‘couple event’ to being in ‘singleness’. Additionally, pandemic-related travel restrictions placed an obstacle for family members to provide postpartum social support. On these grounds, many postpartum women were left feeling isolated, a condition that potentially contributed to a high risk of developing perinatal mood disorders [64,65,66,67,68,69].

Maternal concerns for COVID-19-related restrictions associated with neonate hospitalization (including breastfeeding concerns) were positively associated with maternal depressive symptoms. Τhis confirms evidence showing that mothers with hospitalized neonates in the NICU are more likely to screen positive for postpartum depression [57]. This finding may be explained according to evidence showing that, contrary to the family-centered care model, COVID-19-related restrictions on parental presence in the NICU may inhibit the concept of parents as ‘partners in care’, and limit parent-newborn contact. In addition, early release from the hospital limited access to lactation support. Restrictions may have exacerbated postpartum mothers’ pandemic-related preexisting heightened anxiety with adverse effects on their mental health [70,71,72,73,74,75].

This study contributes to a much-needed area of research. Given the methodological concerns of online data collection of previous studies [76], the current study was based on paper/pencil, well-validated, self-administered questionnaires. The inclusion of both postpartum mothers and fathers adds to a growing body of limited relevant research [13,18,19]. However, some limitations of the study should be taken into account. The small sample size of this study was not representative of the Greek population, and the cross-sectional design precludes establishment of causal relationships. Furthermore, interdependency between participant partners’ perspectives was not investigated. Familial readjustments after the birth of a child affect both members of a couple as a unit. New parents experience similar changes in their daily lives and the work-family conflict is a common stress factor. These associations suggest that a couple’s experience of stress mutually influences one another due to their shared interdependence [50,51,52]. Moreover, we did not examine changes of the same families longitudinally and the possible implications for infant development. Finally, we were unable to make comparisons with a sample of families in the pre-pandemic period.

The findings of the present study suggest that COVID-19-related concerns had a common negative effect on both postpartum mothers’ and fathers’ mental health and on certain aspects of family functioning. The current findings can inform interventions to promote new mothers’ and fathers’ well-being in a timely fashion and improve social support. Health professionals need to be trained to assist in coping with the “spill-over” effects, such as family stress due to financial instability, in future health crisis situations [18,19,21,71].

Note: The Department of Neonatology and the Neonatal Intensive Care Unit (NICU) of the General University Hospital of Heraklion hospitalizes neonates born in Crete and the islands of the South Aegean.

## Figures and Tables

**Table 1:** Descriptive characteristics of the study population.

	N (%) or Mean (SD)		N (%) or Mean (SD)
**Parent characteristics**			
**Gender**		**Parity**	
Women	132 (54.3)	Nulliparous	63 (47.7)
Men	111 (45.7)	Multiparous	69 (52.3)
**Maternal age**	32.94 (5.76)	**Weight gain during pregnancy**	12.49 (5.21)
**Paternal age**	36.60 (6.67)	**Smoking during pregnancy**	
**Maternal origin**		No	124 (93.9)
Urban	91 (68.9)	Yes	8 (6.1)
Rural	41 (31.1)	**Alcohol consumption during pregnancy**	
**Maternal residence**		No	129 (97.7)
Urban	97 (73.5)	Yes	3 (2.3)
Rural	35 (26.5)	**Severe physical problems**	
**Marital status**		No	126 (95.5)
Married	128 (97.0)	Yes	6 (4.5)
Other	4 (3.0)	**Mental health visit**	
**Maternal education**		No	103 (78.8)
Low	5 (3.8)	Yes	28 (21.4)
Medium	49 (37.1)		
High	78 (59.1)		
**Paternal education**		**Child characteristics**	
Low	15 (11.4)	**Gender**	
Medium	60 (45.5)	Male	76 (57.6)
High	57 (43.2)	Female	56 (42.4)
**Maternal occupation**		**Delivery type**	
Public sector	24 (18.2)	Vaginal	29 (22.0)
Private sector	51 (38.6)	Cesarean	103 (78.0)
Self-employed	18 (13.6)	**Type of caesarean**	
Farming	3 (2.3)	Programmed	56 (54.9)
Not working/Housewife	22 (16.7)	Emergency	46 (45.1)
**Paternal occupation**		**Complications during birth**	
Public sector	25 (18.9)	No	124 (93.9)
Private sector	63 (47.7)	Yes	8 (6.1)
Self-employed	36 (27.3)	**Gestational age**	36.19 (2.78)
Farming	6 (4.5)	**Birth weight (g)**	2735.14 (714.29)
Not working	2 (1.5)	**Ever breastfed**	
**Single vs. twin pregnancy**		No	59 (46.8)
Single	110 (83.3)	Yes	67 (53.2)
Twin	22 (16.7)	**Duration of breastfeeding (months)**	1.73 (2.07)

Seventy-six (57.6%) of infants of participant families were males and 56 (42.4%) were females. The mean gestational age was 36.19 (SD = 2.78) weeks. Twenty-nine neonates were born through vaginal delivery (22%), while 103 (78.0%) were born through Caesarean section. More thanhalf of caesareans were elective. According to maternal reports, 58.3% of the newborns were attended, or admitted in the NICU.The mean duration of breastfeeding at the time of assessment was 1.73 (SD = 2.07) months.

**Table 2:** Descriptive statistics for the whole sample and split by gender.

	Total Sample		Females		Males		
	M (SD)	Range	M (SD)	Range	M (SD)	Range	*p*-Value
**FACES-IV**							
**Balanced Cohesion**	30.05 (2.79)	19–35	30.09 (2.67)	23–35	30.01 (2.93)	19–35	0.831
**Balanced Flexibility**	27.53 (3.29)	18–35	27.29 (3.41)	18–35	27.82 (3.12)	20–35	0.224
**Disengaged**	15.03 (4.20)	7–35	15.31 (4.25)	7–35	14.69 (4.13)	8–30	0.260
**Enmeshed**	17.76 (3.86)	9–31	17.59 (4.15)	9–31	17.97 (3.49)	9–49	0.441
**Rigid**	19.42 (3.68)	10–29	19.26 (3.79)	10–29	19.63 (3.54)	12–29	0.451
**Chaotic**	16.48 (3.90)	7–30	16.26 (3.61)	8–29	16.75 (4.30)	7–30	0.335
**Cohesion ratio**	1.92 (0.45)	0.82–3.65	1.92 (0.46)	0.82–3.50	1.93 (0.45)	0.90–3.65	0.820
**Flexibility ratio**	1.58 (0.33)	0.86–2.58	1.58 (0.34)	0.86–2.50	1.57 (0.33)	0.91–2.58	0.865
**Total family functioning ratio**	1.75 (0.34)	1.03–2.89	1.75 (0.37)	1.07–2.63	1.76 (0.35)	1.03–2.89	0.824
**Communication**	42.23 (5.18)	24–50	42.28 (5.27)	24–50	42.16 (5.10)	28–50	0.857
**Satisfaction**	39.73 (5.86)	19–50	39.31 (5.88)	19–50	40.21 (5.83)	23–50	0.240
**Dyadic coping**	143.44 (15.25)	105–172	144.12 (15.53)	105–172	142.47 (14.88)	105–172	0.441
**Perceived social support**	74.00 (8.70)	47–84	74.22 (8.81)	48–84	73.74 (8.59)	47–84	0.676
**Depressive symptoms**	7.90 (5.23)	0–27	8.81 (4.86)	0–21	6.79 (5.47)	0–27	0.003 *
**COVID-19 questionnaire**							
**Daily life**	18.04 (5.16)	(7–31)	18.33 (4.89)	7–29	17.69 (5.45)	7–31	0.338
**Maternal health care**	4.75 (2.27)	(2–10)	4.83 (2.27)	2–10	4.62 (2.27)	2–10	0.508
**Neonate hospitalization**	15.41 (3.98)	(5–25)	15.75 (3.98)	5–24	14.91 (3.96)	7–25	0.263

* *p*-value < 0.005.

**Table 3:** Associations between COVID-19-related life changes and family functioning, dyadic coping, perceived social support and postnatal depressive symptoms in males and females.

	COVID-19 Questionnaire					
	Daily Life		Maternal Health Care		Neonate Hospitalization	
	Males	Females	Males	Females	Males	Females
**Cohesion ratio**	−0.22	−0.23 *	−0.13	−0.06	−0.23	−0.17
**Flexibility ratio**	−0.26	−0.24 **	−0.35 **	−0.01	−0.17	−0.14
**Total family functioning**	−0.27	−0.34 ***	−0.26 *	−0.09	−0.21	−0.18
**Communication**	−0.20 *	−0.22 *	−0.05	−0.17	−0.05	0.02
**Satisfaction**	−0.21 *	−0.29 **	−0.06	−0.14	−0.11	−0.01
**Dyadic coping**	0.06	−0.12	0.02	−0.08	0.02	−0.01
**Perceived social support**	0.10	−0.14	−0.14	−0.21 *	0.06	−0.13
**Depressive symptoms**	0.43 **	0.27 **	0.21	0.23 **	0.06	0.36 **

* *p* < 0.05, ** *p* < 0.01, *** *p* < 0.001.

**Table 4:** Multivariate associations of COVID-19-related life changes with family functioning, dyadic coping, perceived social support and depressive symptoms in parents.

	COVID-19 Questionnaire											
	Daily Life				Maternal Health Care				Neonate Hospitalization			
	Males		Females		Males		Females		Males		Females	
**FACES-IV**	**β**	**95% CI**	**β**	**95% CI**	**β**	**95% CI**	**β**	**95% CI**	**β**	**95% CI**	**β**	**95% CI**
Cohesion	−0.01	(−0.03, 0.00)	**−0.02**	**(−0.04, −0.00)**	−0.02	(−0.07, 0.04)	−0.01	(−0.05, 0.03)	−0.01	(−0.05, 0.03)	−0.02	(−0.06, 0.01)
Flexibility	−0.01	(−0.01, 0.01)	**−0.02**	**(−0.03, −0.00)**	−0.03	(−0.06, 0.00)	−0.01	(−0.04, 0.02)	−0.00	(−0.03, 0.03)	0.01	(−0.01, 0.03)
Total	−0.01	(−0.02, 0.00)	**−0.02**	**(−0.04, −0.01)**	−0.02	(−0.06, 0.02)	−0.02	(−0.04, 0.01)	−0.00	(−0.04, 0.04)	−0.02	(−0.04, 0.01)
Communication	**−0.20**	**(−0.39, −0.00)**	**−0.24**	**(−0.43, −0.04)**	−0.07	(−0.62, 0.47)	**−0.46**	**(−0.88, −0.05)**	−0.06	(−0.48, 0.37)	0.02	(−0.30, 0.35)
Satisfaction	**−0.22**	**(−0.43, −0.01)**	**−0.33**	**(−0.55, −0.12)**	−0.10	(−0.67, 0.47)	−0.45	(−0.91, 0.10)	−0.13	(−0.53, 0.27)	−0.04	(−0.41, 0.33)
**DCI**	0.30	(−0.35, 0.95)	−0.31	(−0.87, 0.25)	0.56	(−1.33, 2.46)	0.29	(−0.92, 1.50)	0.08	(−0.42, 1.78)	−0.02	(−0.97, 0.94)
**MSPSS**	−0.10	(−0.42, 0.23)	−0.25	(−0.58, 0.07)	−0.48	(−1.36, 0.41)	**−0.92**	**(−1.50, −0.24)**	0.16	(−0.58, 0.91)	−0.31	(−0.81, 0.20)
**EPDS**	**0.43**	**(0.24, 0.62)**	**0.27**	**(0.10, 0.45)**	0.41	(−0.13, 0.94)	**0.48**	**(0.10, 0.86)**	0.05	(−0.50, 0.61)	**0.45**	**(0.16, 0.74)**

Abbreviations: EPDS: Edinburgh Postnatal Depression Scale. DCI: Dyadic Coping Inventory; FACES-IV: Family Adaptability and Cohesion Evaluation Scales; MSPSS: Multidimensional Scale of Perceived Social Support. Adjusted β-coefficients and 95% confidence intervals retained from linear regression. Models adjusted for participants’ age, educational level, occupational status, type of delivery, and gestational age. Bold font indicates significant associations (*p* < 0.05).

## Data Availability

The datasets generated during and/or analysed during the current study are available from the corresponding author on reasonable request.
